# 3D Object-Based Card-Sorting: A Method for Eliciting Multimodal Reasoning in Chemistry

**DOI:** 10.1021/acs.jchemed.5c00638

**Published:** 2025-10-03

**Authors:** Robin Morgenstern, Samuel Pazicni, Sarah A. Swineheart, Maia Popova

**Affiliations:** † Department of Chemistry, 5228 University of Wisconsin—Madison, Madison, Wisconsin 53706, United States; ‡ Department of Chemistry & Biochemistry, 14616 University of North Carolina at Greensboro, Greensboro, North Carolina 27412, United States

**Keywords:** General Public, First-Year Undergraduate/General, Second-Year Undergraduate, Upper-Division Undergraduate, Chemistry Education Research, Inorganic Chemistry, Hands-On Learning/Manipulatives, Testing/Assessment, Molecular Properties/Structure

## Abstract

This contribution introduces 3D object-based card sorting as a novel method for eliciting and analyzing students’ multimodal reasoning in chemistry. Building on traditional card sort methodologies, this approach incorporates manipulable molecular models (either physical or virtual) to explore how students reason about spatially complex concepts, such as molecular symmetry. We describe the task design, illustrate its potential through sample student excerpts, and evaluate its methodological integrity using the *Journal Article Reporting Standards for Qualitative Research in Psychology*. The sorting interviews generated rich, multimodal data, including gestures, model manipulation, and verbal reasoning. While the method captures fine-grained, process-level reasoning, it also affords insights at a coarser grain size, supporting inferences about students’ conceptual and epistemological resources as they categorize. This work demonstrates how 3D object-based card sorting can make students’ reasoning more visible and analyzable, offering new opportunities for research on spatial thinking, representation use, and embodied cognition in chemistry. We conclude by outlining implications for both research and classroom applications.

## Graphical abstract

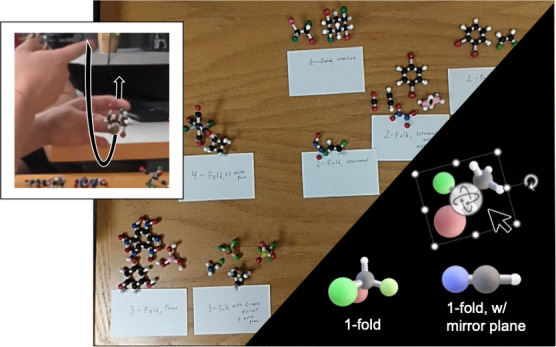

Card sorting is a versatile research method used to investigate how individuals organize, relate, and interpret conceptual information. In its most basic form, participants group items (often presented on cards) based on perceived similarity or shared attributes. This method has been employed across diverse fields (including cognitive psychology, education, and human-computer interaction) to explore how people structure conceptual knowledge and understand relationships among ideas.

In seminal work using card sorting, Chi and colleagues contrasted expert and novice characterizations of physics problems, showing that the resulting groupings reflected differences in conceptual understanding.^1^ While such studies demonstrate the value of analyzing final sorts to reveal how participants structure knowledge, they often do not offer insight into the reasoning processes that generate those groupings. In response, hybrid approaches have emerged that embed card sorting within interviews, using the activity not only as a data source but also a conversational structure. Conrad and Tucker describe these methods as enabling participants to articulate their thinking while sorting, offering researchers a window into how decisions evolve though reflection, revision and dialogue.^2^ This makes such approaches especially valuable for investigating abstract ideas, common in disciplines like chemistry.

This paper introduces a methodological innovation: 3D object-based card sorting, in which participants sort manipulable molecular models (either physical or virtual). We frame this as a methodological contribution to chemistry education research (CER), where traditional card sorting tasks have typically relied on static two-dimensional images. Our aim is to demonstrate how incorporating 3D representations can elicit richer, multimodal forms of reasoning and expression (encompassing talk, gesture, and manipulation) not accessible through traditional card formats alone.

We present this method using molecular symmetry as a test case—not as a central research focus, but to illustrate the method’s potential. In what follows, we situate our approach within prior CER card sort literature, describe the theoretical and design commitments that informed its development, and offer a rationale for its broader use. We then detail how the method was implemented in interviews exploring students’ reasoning about symmetry and highlight the kinds of insights it affords. In particular, we show how this approach enables the collection and analysis of multimodal reasoning-in-action, capturing not only verbal explanations, but also gestures, model manipulation, and interpretive decisions as they unfold. We conclude with guidance for adapting the method and discuss its potential applications in both CER and learning environments.

## Card Sorting as a Methodology in Chemistry Education Research

Card sorting has emerged as a useful methodology in CER, used to investigate how students, instructors, and preservice teachers organize disciplinary knowledge, articulate teaching beliefs and orientations, and engage with representational modes.^3–22^ Across the literature, however, implementations vary widely in both structure and purpose. Some studies rely primarily on participants’ final groupings to infer conceptual or pedagogical structures, while others embed the activity within interviews or think-aloud protocols to foreground the reasoning processes behind those groupings. This variation makes it difficult to synthesize findings across studies or to articulate the methodological contributions of different card sort designs.

Card sorting tasks are often described along two structural dimensions: *open versus closed* (whether participants generate their own categories or use predefined ones) and *moderated versus unmoderated* (whether a facilitator is present to observe or prompt the sorting process). While this typology provides useful terminology for describing task formats, it does not fully capture the epistemic orientation of the study—that is, the role the method plays in making participants’ thinking visible, or how it supports research aims.

Rather than classifying card sorting tasks solely by format, we found it more productive to organize studies according to how sorting is used to elicit and analyze reasoning. We draw on a framework inspired by Conrad and Tucker, who describe card sorting as a tool that can structure participant reflection and dialogue.^2^ Based on this orientation, we classify CER studies using card sorting into three types, distinguished by how the method is embedded in the research design and the kind of reasoning it aims to elicit:

- **Outcome-Focused Sorting**. Final groupings are analyzed as stand-alone artifacts, without embedding the task in a conversational or reflective context.
- **Reflective Post-Sort Interviews**. Sorting prompts retrospective conversation. Participants are asked to explain and justify their groupings after completing the task.
- **Real-Time Reasoning During Sorting**. Sorting is embedded in a live, conversational context. Participants verbalize their thinking while sorting, making in-the-moment reasoning visible.

This three-part typology emphasizes the analytic function of card sorting and provides a way to compare studies based on their data sources, research goals, and assumptions about cognition.

### Outcome-Focused Sorting

Studies in this category treat final card sorting groupings as diagnostic artifacts. These designs focus on *what* was sorted rather than *how*, omitting participant interaction or explanation during or after the task. For example, Krieter and colleagues designed a card-sorting task to assess whether students were learning to “think like chemists.”^10^ Participants sorted chemistry problems, and their groupings were analyzed statistically to identify whether they reflected “deep” conceptual structures or “surface” features like terminology or visual layout. The study introduced a quantitative metric to assess participant groupings with expert and novice norms, offering a way to track conceptual development over time. Similarly, den Otter et al. developed a structure–property reasoning task in which students sorted items and created maps.^20^ The final products were analyzed to compare reasoning between preuniversity and first-year students. Sizemore et al. extended this approach by incorporating machine learning to examine student-written justifications alongside card groupings, revealing shifts in categorization strategies associated with increasing expertise.^21^

### Reflective Post-sort Interviews

These studies use sorting to elicit retrospective explanation, treating the task as a springboard for participants to articulate their reasoning after the fact. In CER, many studies adopting this structure build on work by Friedrichsen and Dana^23,24^ using card sorting to examine how instructors conceptualize teaching.^8,12,14,15,17^ For instance, Akın and Uzuntiryaki-Kondakci asked teachers to sort scenario cards representing different orientations (didactic vs inquiry based) and then explain their groupings.^14^ Other studies explore how participants stated beliefs align with reform-oriented practices by prompting them to justify their sort categories.^3,7^

Still others focus on representational reasoning. Head and colleagues had preservice chemistry teachers sort image cards aligned to Johnstone’s triangle,^25,26^ then used interviews to probe how participants interpreted representational levels.^13^ Kelly et al. used a card sort to investigate epistemological resources activated when students evaluated submicroscopic representations of chemical reactions.^18^ Irby et al. examined participants ability to coordinate understanding across Johnstone’s three levels.^9^

Some researchers focus on representational reasoning beyond analysis through the lens of Johnstone’s triangle. Kozma and Russell asked students to sort static image cards and used interviews to explore how they interpreted visual representations.^4^ Domin et al. tracked how categorization strategies shifted during learning by asking students to reflect on their grouping of organic molecules.^5^ Galloway et al. similarly used postsort interviews to investigate how students categorized organic mechanisms, focusing on interpretation of curved arrows and transformation patterns.^16^

Together, these studies use card sorting to generate artifacts for reflection, allowing researchers to infer conceptual, pedagogical, or representational frameworks. However, because reasoning is elicited *post hoc*, these approaches do not capture real time sensemaking during the act of sorting.

### Real-Time Reasoning During Sorting

This final category positions card sorting as a generative activity, in which participants reason aloud while making categorization decisions. Such studies aim to capture reasoning-in-action and trace how conceptual understanding unfolds during the task. Stains and Talanquer prompted both undergraduate and graduate chemistry students to think aloud while sorting symbolic and submicroscopic reaction representations, revealing differences in the features participants emphasized.^6^ Graulich and Bhattacharyya used conversational interviews to examine how students grouped organic reaction mechanisms, focusing on how reasoning developed through dialogue.^11^ Robinson et al. used real-time prompts to study how students interpreted electrostatic potential maps in sorting tasks.^22^

Despite these advances, no study in this category has explicitly examined gesture or physical model manipulation as data. Most card sorting designs rely on static 2D representations and treat verbal or written responses as the primary evidence of reasoning. Even in real-time contexts, embodied dimensions of thinking (such as how students manipulate objects or gesture toward features) remain underexplored. This limits what we can observe about how students coordinate spatial, conceptual, and representational reasoning in chemistry.

## Methodological Contributions: Addressing Representational and Spatial Challenges

These gaps in the literature came into sharper focus as we set out to investigate how students reason about molecular symmetry, a domain thought to present distinctive representational and spatial challenges.^27,28^ While traditional card sorting methods have been used effectively to examine categorization and conceptual structure, their reliance on static, 2D representations and verbal explanation limited our ability to observe the kinds of multimodal reasoning we hoped to document. Specifically, we were interested in reasoning that involves rotating molecules mentally or physically, tracing imagined symmetry elements, and enacting symmetry operations that are often difficult to express verbally.

To address these limitations, we developed a 3D object-based card sorting methodology that invites students to work with manipulable molecular models and to articulate their thinking through open-ended, conversational interviews. This design supports reasoning-in-action by capturing students’ gestures, model manipulations, and spontaneous explanations as they work through the task in real time. Rather than focusing solely on sorting outcomes or *post hoc* justifications, our approach centers the sorting process itself as a rich site for observing sensemaking.

Although this method was initially designed to study reasoning about symmetry, it offers broader utility for investigating sensemaking in structurally rich domains where spatial reasoning, representation use, and embodied engagement are central. The combination of structural models, open-ended prompts, and multimodal data collection affords insight into students’ reasoning at multiple grain sizes, from fine-grained interactional moves to broader conceptual and epistemological orientations. In the next section, we articulate the theoretical commitments that informed this design, including our assumptions about the nature of reasoning, the role of representations in learning, and the value of multimodal data for interpreting student thinking.

## Theoretical Commitments Guiding the Design of the 3D Object Sorting Task

The design of our 3D object-based card sorting methodology draws on five complementary theoretical commitments that shape how we support students in expressing their thinking—by allowing them to speak, gesture, and physically interact with molecular models as they work through ideas in real time. In contrast to many card sorting approaches that treat outcomes as static indicators of conceptual understanding, we view sorting as a generative context, one that elicits situated reasoning and supports multimodal sensemaking. This stance guides both how we designed the sorting activity and how we interpret participants’ reasoning during the task.

**Working memory** ^29^ limitations and principles from **cognitive load theory** ^30^ support our commitment to designing a sorting environment that invites reasoning-in-action by reducing unnecessary cognitive demands. According to cognitive load theory, effective learning environments reduce extraneous cognitive load (effort that does not support learning) while fostering germane processing (effort devoted to constructing and refining mental models).^31^ Tasks that require students to mentally reconstruct 3D spatial relationships from 2D diagrams can consume substantial working memory resources, limiting capacity for meaningful reasoning.

To address this, our design makes molecular structure and symmetry features perceptually available and manipulable by providing 3D models. This reduces the need for mental rotation and eliminates unfamiliar representational elements, which can impose unnecessary processing demands.^32^ Instead of mentally simulating symmetry operations, students can engage with them physically, offloading some spatial reasoning to the perceptual-motor system. This approach aligns with cognitive load theory recommendations to distribute processing across modalities and to match task demands to learners’ capacities.^31^

CER reinforces this principle: physical models can lower representational barriers, reduce cognitive demands, and support engagement with chemical ideas.^33,34^ In our methodology design, exploratory manipulation of models was not a distraction but a central feature of students’ sensemaking, consistent with the idea that cognitive load should be managed (not minimized) to allow space for meaningful conceptual engagement to emerge.^31^ Thus, we designed the task to help students offload unnecessary mental effort (especially the kind caused by having to mentally rotate or interpret 2D diagrams) by giving them 3D models they could see and manipulate directly.

**Representational competence** refers to a learner’s ability to interpret, generate, and translate among different forms of scientific representations in support of conceptual reasoning.^4,35^ In chemistry, this includes fluency with diagrams such as wedge-dash structures, line-angle formulas, and molecular models, each encoding spatial information in distinct visual conventions. While representational fluency is an important instructional goal, research has shown that students frequently struggle to coordinate 2D and 3D forms,^33,36–38^ and that their reasoning can vary across different representations,^39^ all of which can potentially confound interpretation of card sort data.

Our methodological design aimed to reduce these representational barriers by relying exclusively on 3D ball-and-stick models. This decision eliminated the need for students to interpret or mentally reconstruct molecular structures from abstract 2D diagrams, making spatial features such as symmetry elements more directly accessible and enabling participants to focus on classification and conceptual reasoning. As Schönborn and Anderson emphasize, the interpretability of visual representations depends not only on prior knowledge but also on the cognitive demands imposed by the representational form itself.^36,37^ By avoiding representational conventions that are unfamiliar or potentially misleading, our task design supports clearer elicitation of students’ ideas about molecular symmetry.

**Knowledge-in-Pieces** ^40^ is a family of theoretical models^41^ that frame knowledge as composed of many fine-grained, context-sensitive elements, activated dynamically in response to specific tasks and environments. Rather than assuming that learners hold coherent conceptions or stable misconceptions, this framework views reasoning as emergent and situated, shaped by prior experiences, local context, and the representational tools at hand.^42^ In the domain of molecular symmetry student reasoning may draw not only on formal instruction, but also on spatial intuitions,^43–46^ aesthetic familiarity,^47^ and disciplinary or cultural cues.^48–50^ Studies have also shown that students’ identification of symmetry elements can be sensitive to features such as orientation of the molecular orientation,^51,52^ highlighting the importance of studying symmetry sensemaking in context.

Traditional card sort studies often treat final groupings as stable reflections of conceptual structure or levels of expertise. This interpretation is not well aligned with a Knowledge-in-Pieces perspective. However, when sorting is embedded within a conversational context (where participants explain and reflect on their thinking), card sorting can serve as a generative environment for observing reasoning-in-action. Recent studies by Kelly et al.^18^ and Robinson et al.^22^ demonstrate how card sorting, when framed appropriately, can illuminate how students activate, coordinate, and reorganize conceptual resources. Our approach builds on this work by leveraging multimodal data (including gesture, model manipulation, and talk) not merely to classify thinking, but to understand how students make sense of chemical structure across the task.

**Epistemological framing** refers to how individuals interpret the nature of the knowledge-building activity they are engaged in—that is, their sense of what kind of thinking the task calls for.^53^ These framings influence how participants engage with the task and the types of ideas they make visible, and may change throughout the task depending on the participants’ perceptions of the task or cues from the interviewer. Drawing on Russ et al.’s work on cognitive interviews,^54^ we recognize three broad framing patterns that students may adopt at different points throughout a task:

- a *sensemaking frame*, in which they explore and develop ideas;
- an *oral examination frame*, in which they attempt to recall or perform known information for an evaluator; and
- an *expert interview frame*, in which they present themselves as knowledgeable and definitive.

Each of these framings supports different epistemic stances and reasoning behaviors.

Because our goal is to elicit students’ emergent, context-sensitive reasoning, the framing students adopt has direct implications for the quality and nature of data we can observe. Students operating in an oral exam frame may restrict their responses to rehearsed facts, while those in a sensemaking frame are more likely to externalize tentative, emergent ideas. To promote the latter, our design incorporated open-ended prompts, manipulable 3D models, and a conversational interview structure—scaffolds intended to position participants as active thinkers rather than passive respondents, consistent with Russ et al.’s conception of framing as dynamically cued by context. Additionally, the interview protocol included guidance that the interviewer should redirect participants who appeared to be focused on correctness or terminology by reassuring them that there were no correct answers to this task and that the goal was simply to understand how the participants were thinking in the moment.

Lastly, **gesture and embodied interaction** are treated not as peripheral behaviors but as central to students’ reasoning. Research in cognitive science and mathematics education shows that gestures can reveal dimensions of spatial and conceptual thinking that are not always captured in speech.^55,56^ In chemistry, disciplinary language often relies on abstract symbols and technical vocabulary that can obscure students’ ideas,^57^ particularly in domains like molecular symmetry, where verbal explanations may be less accessible. In such contexts, gestures and model manipulation may serve as more intuitive forms of communication and meaning making.^58^

Our sorting task was deliberately designed to support multimodal expression, allowing participants to point, gesture, and physically manipulate models. These embodied actions helped externalize imagined transformations, highlight spatial features, and support reasoning that often exceeds what participants can express verbally. By capturing these embodied dimensions of thought through video and observation, our method offers access to cognitive processes that traditional language-based methods may miss.

### How Theoretical Commitments Shaped Task Design

Together, these theoretical commitments support a methodological stance in which reasoning is multimodal, situated, and emergent. Our use of card sorting departs from traditional schema-driven approaches that treat the task as a static assessment of conceptual structure. Instead, we treat sorting as an interactive space for observing how students construct meaning through gesture, talk, and action. This orientation contrasts with studies that use card sorting primarily to distinguish novices and experts based on final sort outcomes.^1,10,59–61^ Instead, we align with Conrad and Tucker’s conception of card sorting as a “conversational space”,^2^ emphasizing participants’ interpretive work and treating sorting as a site for idea generation, revision, and embodied sensemaking.

## Design and Implementation of the 3D Card Sorting Task

We developed and deployed our 3D object-based card sorting method to investigate how undergraduate students reason about molecular symmetry. While the design was tailored to this domain, the method itself is broadly applicable to other topics where chemical structure, spatial reasoning, and multimodal engagement play a central role.

To enable participation across institutional boundaries and preserve opportunities for gesture and embodied reasoning, we designed the task to be usable in both face-to-face and remote interviews. Participants worked with manipulable 3D molecular models, either as physical objects or within an interactive digital platform, allowing them to express their thinking through gesture, object manipulation, and talk. This decision was influenced by practical considerations (i.e., the need to conduct interviews remotely with geographically distributed students) as well as by a growing body of work highlighting the methodological potential of virtual, interactive tasks.^62,63^ While physical models afford richer tactile engagement, digital models enabled broader participation and preserved core features of manipulability. This parallel design reflects best practices for ensuring methodological consistency in remote and in-person interviews.^64^ It also supports credibility^65^ by allowing us to examine whether participants engaged the task similarly across settings—a point we return to later. Importantly, this design choice foreshadowed later analyses comparing student engagement across modalities, not to assess equivalence *per se*, but to examine how physical and virtual settings may shape epistemological framing and the kinds of reasoning made visible.

In the sections that follow, we detail our design decisions (including model selection/construction, task materials, prompt structure, and interview format) and explain how each choice aligns with our theoretical commitments to eliciting reasoning-in-action through embodied and exploratory engagement.

### Model Selection and Construction

We selected 25 molecular models representing a range of point groups and varying levels of complexity (Table 1). These models were deliberately designed to minimize perceptual and structural variability and maintain consistency across participants. We avoided commercial model kits, which often include rotatable bonds and thus, conformational flexibility. Such flexibility could lead to participants altering the prescribed structure and sorting different conformers during the task, complicating comparisons across interviews. Instead, we used fixed conformers, carefully constructed to control molecular orientation and ensure that all participants encountered identical spatial structures.

**Table 1:**
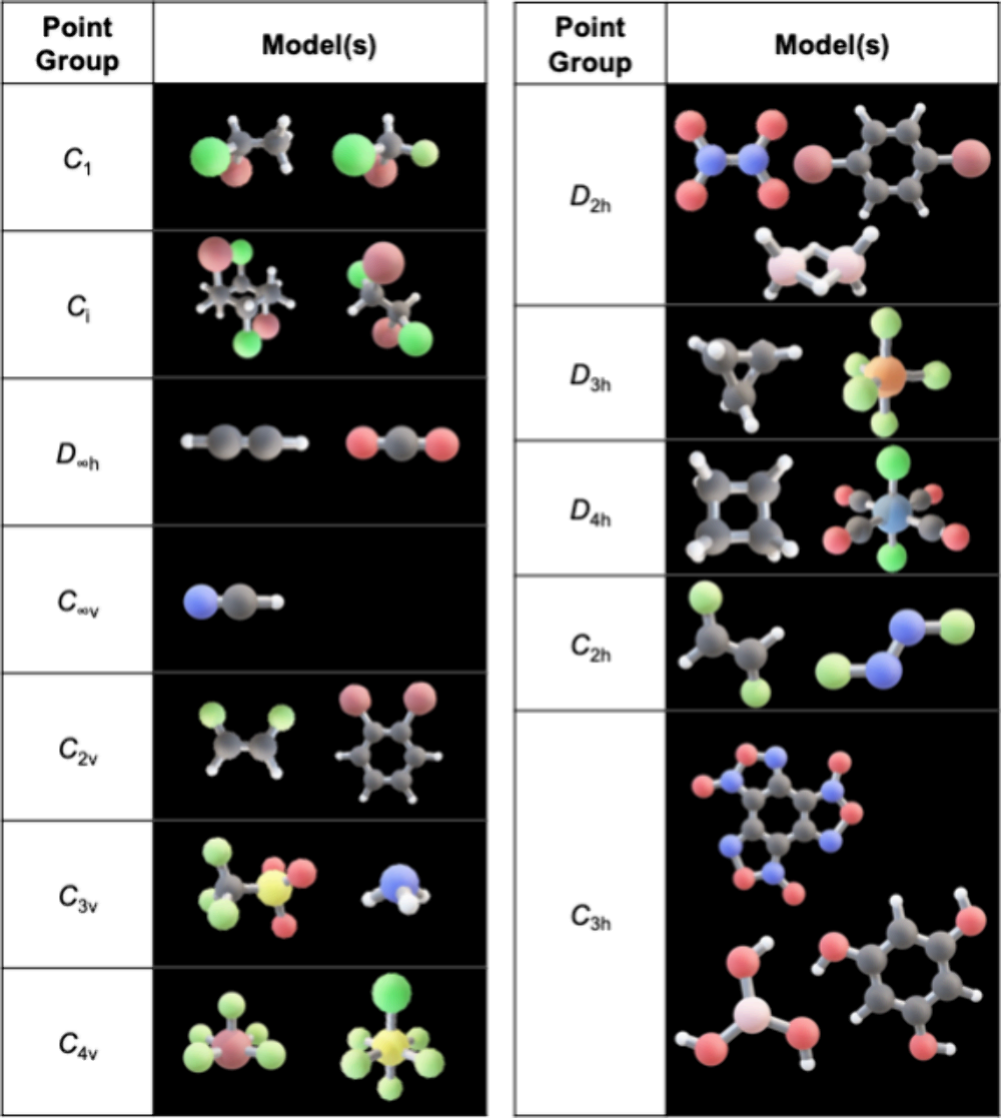
Set of Molecular Structures Used in the Sorting Task, Selected to Represent Diverse Point Groups and Symmetry Features Relevant to Eliciting Student Reasoning

The physical models were first built in Chem3D, which allowed us to draw 2D bond-line structures and convert them to 3D structures. Most molecules were generated in a default low-energy conformation, while the coordinates of cyclobutene were manually adjusted to ensure that all four carbon atoms lay in the same plane. These Chem3D structures were exported as PDB files and converted to STL files using Mercury. The STL files were then imported into PrusaSlicer and printed on an Ender3 3D printer using white PLA filament. All PDB files are provided as . After printing, each model was sanded smooth, primed, and hand-painted with standard atomic color conventions (e.g., black for carbon, white for hydrogen, red for oxygen, etc.) to match the appearance of conventional molecular model kits, reinforcing visual familiarity.

Virtual versions of these models were generated in Blender using the ″Atomic Blender PDB/XYZ″ add-on, which allowed us to render the same PDB files used for the physical prints. Bond lengths, thicknesses, atom radii, and colors were adjusted to closely match the appearance and proportions of the physical models. The resulting OBJ and MTL files were embedded into a PowerPoint slide as manipulable 3D objects, enabling participants to interact with the models directly on their own devices. This PowerPoint file is provided as .

Our design aimed to ensure consistency across physical and digital modalities while maximizing accessibility and usability across participant hardware and skill levels. By fixing conformations and standardizing visual features, we minimized representational ambiguity and perceptual variability. This consistency allowed us to examine how the form of the models (physical or virtual) might influence students’ engagement, reasoning, and epistemological framing of the sorting task. Would participants approach the task as a test of correctness, or as an opportunity to explore? Our dual-format design preserved structural comparability while allowing us to probe such epistemological framing differences.

### Interview Task and Prompt Structure

The interviews followed a semistructured protocol consisting of three phases: (1) pretask discussion, (2) card sorting task, and (3) post-task reflection. The card sort prompt was intentionally open-ended. We asked participants to sort molecules “based on the concept of symmetry,” without prescribing a specific approach. This design reflected our theoretical commitments. Traditional symmetry tasks often ask students to identify elements (e.g., planes of symmetry or rotation axes) or assign point groups, framing symmetry as a technical exercise. In contrast, our open-ended prompt positioned symmetry as a conceptual lens rather than a solved problem. From a Knowledge-in-Pieces perspective, this created an interview environment in which reasoning could emerge dynamically through interaction with the models and conversation with the interviewer, surfacing diverse knowledge elements grounded in instruction, intuition, and disciplinary or aesthetic experience. This framing was intended to elicit a sensemaking orientation, empowering students to question, explore, and revise their ideas rather than perform correctness.

Each interview session lasted approximately 1 h and was audio- and video-recorded for subsequent analysis. In face-to-face interview sessions, an overhead camera captured molecular groupings and movements, while a side-view camera recorded gestures (Figure 1). In virtual sessions, screen-recording software captured participants’ interactions with digital models embedded in PowerPoint, while a thumbnail webcam view documented their gestures (Figure 2).

**Figure 1:**
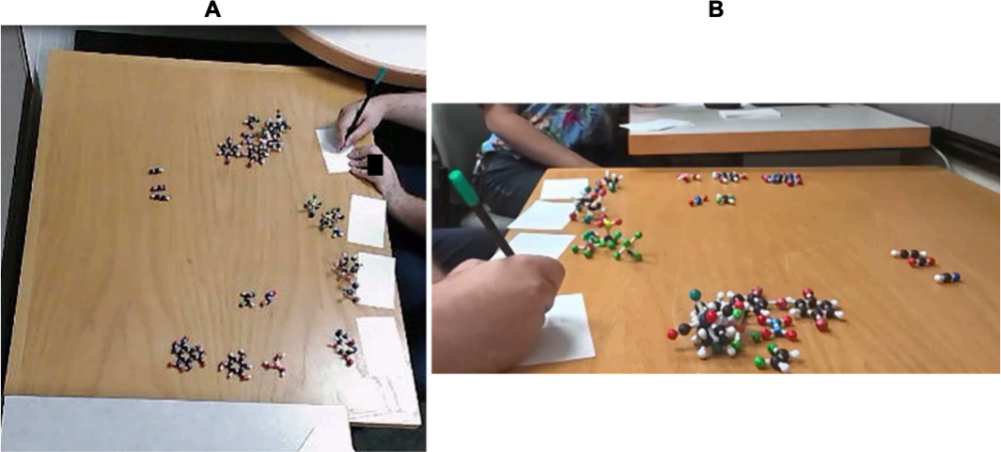
In-person interview setup illustrating data capture from two angles. (A) Overhead view showing molecular model layout and sorting surface; (B) side view capturing participant gestures and model interactions. This dual-camera configuration enabled precise documentation of students’ reasoning beyond verbal communication. Identifying features such as jewelry have been obscured for participant anonymity.

**Figure 2:**
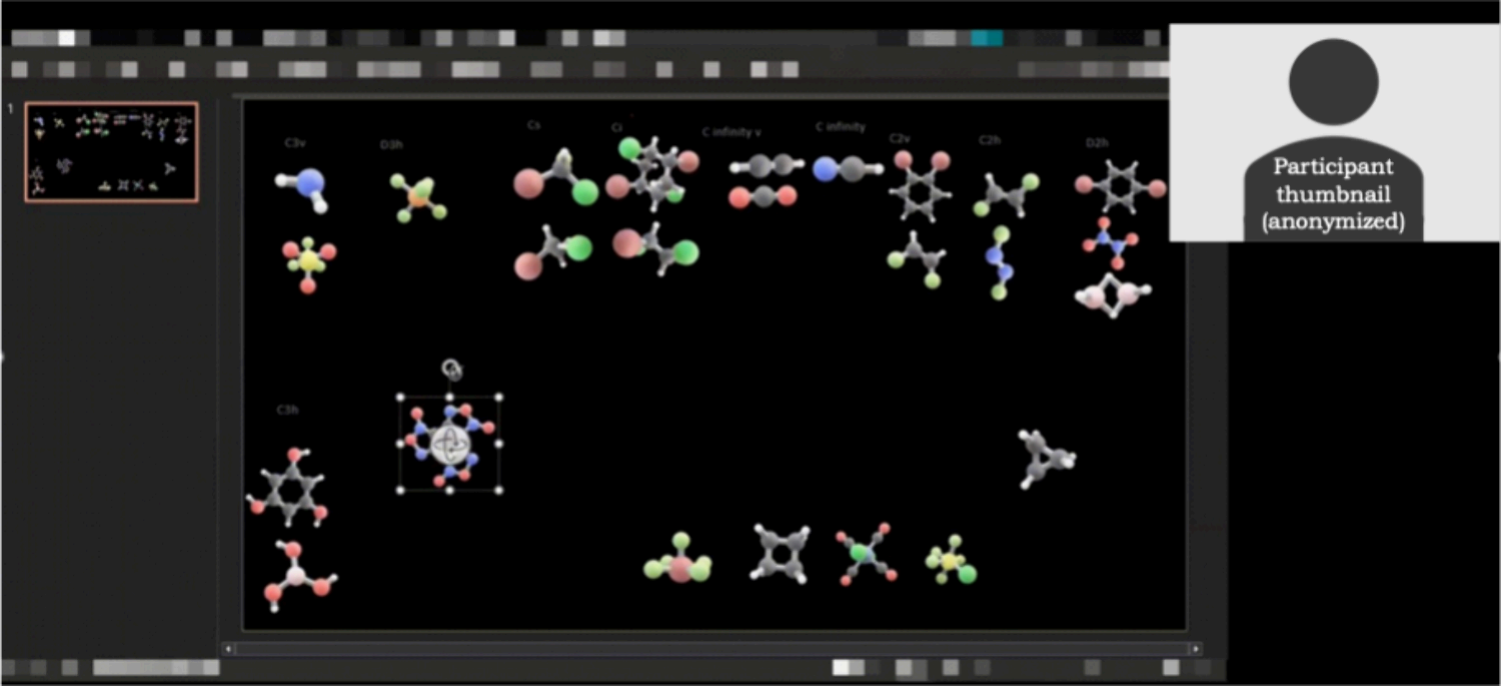
Virtual interview setup conducted over Zoom. Screen-recording software captured participant interactions with digital 3D models embedded in PowerPoint, while a thumbnail webcam view recorded hand gestures. This configuration supported multimodal analysis by integrating screen-based model manipulation with physical gesturing, allowing comparisons to in-person sessions.

#### Pre-task Discussion

Participants were asked to provide a list of chemistry courses they had taken in the past. They were asked which of those chemistry courses used the concept of symmetry, and if any other courses they had taken outside of the field of chemistry had used the concept of symmetry.

#### Card Sorting Task

Participants were asked to “sort these 25 molecules into categories based on the concept of symmetry while thinking out loud.” Participants were informed that they could create as many or as few categories as they wished, and that overlapping categories were acceptable. They were also informed that they would be asked to give their categories labels that describe what the molecules in that category have in common, and that they could choose to label their categories as they sort or at the end of the sorting process, whichever felt more natural to them. They were encouraged to handle, rotate, and manipulate the models as needed. During sorting, the interviewer primarily adopted a responsive role, offering follow-up prompts only when participants’ utterances were unclear or ambiguous. Additional clarifying questions were asked during prolonged silences or after multiple model manipulations to encourage reflection (e.g., “Can you say what you’re thinking as you handle that one?”). These real-time prompts were not simply clarifying questions but part of our effort to frame the card sorting task as a conversational space,^2^ enabling participants to articulate, revise, and expand their reasoning through dialogue. By treating the sorting task as an unfolding sensemaking activity rather than a fixed performance, we aimed to capture the emergent, situated nature of their reasoning.

#### Post-task Reflection

Participants were asked clarifying questions about category labels and inclusion criteria. They were also asked if any of the categories were more closely related to each other than they were to other categories. Occasionally, participants were asked to elaborate on their sorting decisions, reflect on challenges, and discuss their strategies for identifying symmetry elements. Rather than treating post-task reflections as validation of accuracy, we treated them as further windows into participants’ evolving sensemaking.

## Methodological Integrity

In qualitative paradigms, methodological rigor is best understood not through replicability or generalizability, but through the coherence of a study’s design with its goals and theoretical commitments. The *Journal Article Reporting Standards for Qualitative Research in Psychology* (JARS-Qual)^66^ offers the construct of *methodological integrity* to guide such evaluations. Developed through extensive consultation across qualitative traditions, methodological integrity foregrounds the alignment of methods with both the subject matter and the research aims, with an emphasis on how methodological decisions are enacted and sustained throughout the research process.

Levitt et al.^66,67^ describe methodological integrity as composed of two interrelated processes:

1. **fidelity to the subject matter**, or the extent to which procedures develop and maintain allegiance to the phenomenon as it is understood within the study’s paradigm; and
2. **utility in achieving research goals**, or the degree to which methods effectively support the analytic aims of the study.

Together, fidelity and utility provide a foundation for evaluating whether a study’s procedures afford meaningful insight into the phenomenon of interest.

In our work, the phenomenon of interest is student reasoning about molecular symmetry, reasoning that is often multimodal, emergent, and deeply embedded in spatial interaction. Our 3D object-based card sorting task was designed to elicit and make visible this reasoning. Below, we provide evidence that the method fulfills both components of methodological integrity, using observed participant behaviors as primary evidence.

### Participants

Thirty students participated in this work: six in in-person interviews and 24 in virtual interviews. The in-person group included two general chemistry students with no formal instruction on molecular symmetry and four inorganic chemistry graduate students who had completed at least one graduate-level course on the topic. All six students were from the same institution and participated under an IRB-exempt protocol (2022–0714) approved by the University of Wisconsin–Madison Minimal Risk Research IRB. At the start of the interview, the consent process was reviewed, and participants confirmed their agreement by signing a physical consent form.

The virtual group consisted of 24 undergraduate students enrolled in inorganic chemistry courses at 12 different institutions. Each had previously indicated, during preinstruction consent, willingness to participate in follow-up interviews. Recruitment occurred after instruction and assessment on molecular symmetry had concluded, ensuring content exposure. Due to low response rates, all students who had provided consent were invited, and all who responded were interviewed. These interviews were conducted under a separate protocol reviewed by the University of Wisconsin–Madison Health Sciences IRB (2022–0248), which determined that continuing review was not required. Consent materials were emailed to participants 3 days prior to the interview. At the beginning of the interview, the consent process was revisited, and participants were asked to confirm that they had reviewed the information and agreed to participate. Digital consent was documented using Adobe Acrobat or a similar secure electronic signature platform.

### Fidelity to the Subject Matter

Fidelity refers to the alignment between data collection procedures and the nature of the phenomenon under study, as understood within the research’s theoretical and methodological commitments. In our case, this meant developing methods that captured students’ in-the-moment reasoning about molecular symmetry through verbal, embodied, and interactive forms of expression.

Consistent with the *JARS-Qual* guidelines and Levitt et al.’s framework, we support fidelity through (a) data adequacy (collecting data that reflect relevant variations in student reasoning); (b) perspective management (attending to the influence of our own perspectives on data collection and analysis); and (c) groundedness (ensuring that claims are supported by observable behavior).

To support data adequacy, we designed the task in line with a typology of card sort methodologies developed through our literature review. Rather than using final sorts alone, we embedded sorting within interviews as a conversational structure to foreground emergent reasoning. The task incorporated manipulable 3D models (either physical or virtual) that enabled gesture, rotation, and reinspection. This design fostered a range of context-sensitive reasoning strategies, including a behavior we term *exploratory rotation*, where students rotated a model continuously while articulating evolving ideas. This was distinct from purposeful movements, such as rotating a molecule to communicate an idea or performing a deliberate symmetry operation.

These embodied actions were not merely expressive but formative. Students rotated, aligned, and inspected models while gesturing to simulate symmetry elements, sometimes with their hands, sometimes with drawing tools. Simultaneously, participants verbalized their observations and classifications in real time. This co-occurrence of gesture, manipulation, and talk enabled access to a wide range of knowledge elements: some explicitly stated (e.g., “this molecule has a *C*_3_ axis”), others inferred from behavior (e.g., assigning a molecule to a group labeled “linear”), and still others revealed through talk (e.g., “I’m spinning this one to look for a rotational axis”), and embodiment (e.g., tracking atomic movement with a finger while rotating the model). The result was a record of students’ multimodal reasoning.

We observed strong methodological consistency across settings. Whether using fingers or cursors, paper or PowerPoint tools, participants in both in-person and virtual sessions demonstrated comparable behaviors (Figure 3). These cross-setting similarities reinforce that the method accessed the same phenomenon (context-sensitive, embodied reasoning) regardless of implementation format.

**Figure 3:**
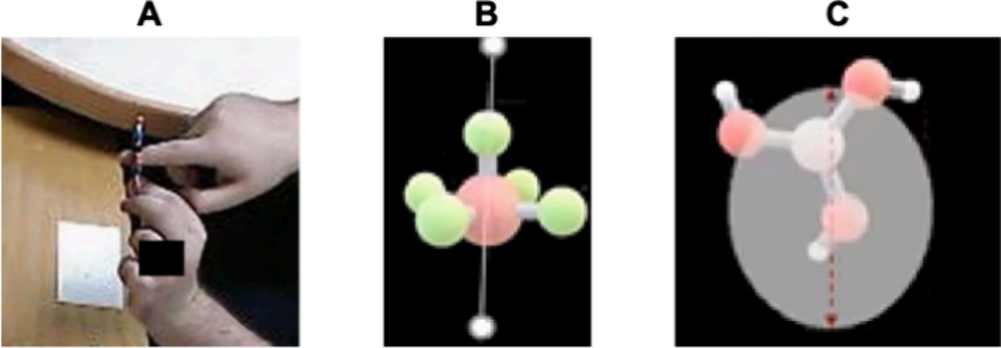
Student gestures illustrating symmetry concepts across physical and virtual interview formats. (A) In-person participant using their finger to demonstrate the position of a rotational axis on a physical model; (B) virtual participant using PowerPoint drawing tools to represent a rotational axis; (C) virtual participant using drawing tools to represent a mirror plane. These examples highlight how the 3D object-based sorting method elicits gesture-augmented reasoning in both physical and digital environments. Identifying features have been obscured to maintain anonymity.

Our transcription and analysis practices further supported groundedness and perspective management. Transcripts integrated speech, gesture, and model manipulation. Multicam recordings (face-to-face interviews) and screen/webcam capture (virtual interviews) documented gesture and spatial orientation. Figure 4 presents a representative excerpt illustrating how multimodal behaviors were captured. A second coder independently reviewed selected videos and transcripts to refine gesture annotations in an iterative, collaborative process. These annotations were finalized before formal coding, helping mitigate interpretive bias and strengthen grounding of claims.

**Figure 4:**
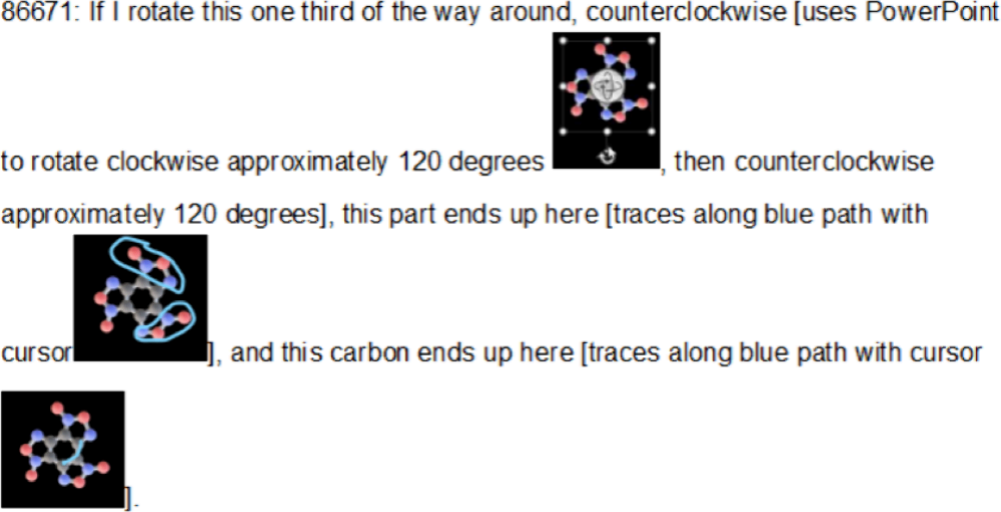
Example of a transcript excerpt from virtual interview. In this example, a student is engaging with the molecule benzotrifuroxan. The transcript is annotated with both verbal descriptions of their gestures (e.g., “traces along blue path with cursor”) as well as screen captures of molecular orientation and student inscriptions upon the molecule.

### Utility in Achieving Research Goals

Utility reflects the extent to which a study’s methods enable meaningful insight aligned with its analytic goals. In this methods-focused paper, our goal is not to generalize findings about student understanding, but to demonstrate the value of a 3D object-based card sort for eliciting and analyzing reasoning in real time. Following Levitt et al., we support utility through: (a) contextualization of data (recognizing how research settings influence participant responses); (b) catalyst for insight (designing methods that elicit rich, analyzable reasoning); (c) meaningful contributions (producing data that advance understanding of the phenomenon); and (d) coherence among findings (attending to participant variation as methodological affordance, not inconsistency).

To support contextualization, we used a common task prompt and materials across settings and recruited students with varied backgrounds. The open-ended prompt asked students to sort molecules “based on the concept of symmetry,” positioning symmetry as a conceptual lens rather than a problem with a known solution. This framing encouraged reflection on reasoning, not recall.

The task served as a catalyst for insight by inviting students to test and refine their ideas through interaction. As Figure 5 illustrates, participants engaged in dynamic processes of noticing, analogy, and verification—for example, comparing a molecule to NH_3_, inferring symmetry properties, and then using stepwise rotation and annotation to confirm. Such sequences would be difficult to observe using methods that rely solely on final categorization.

**Figure 5:**
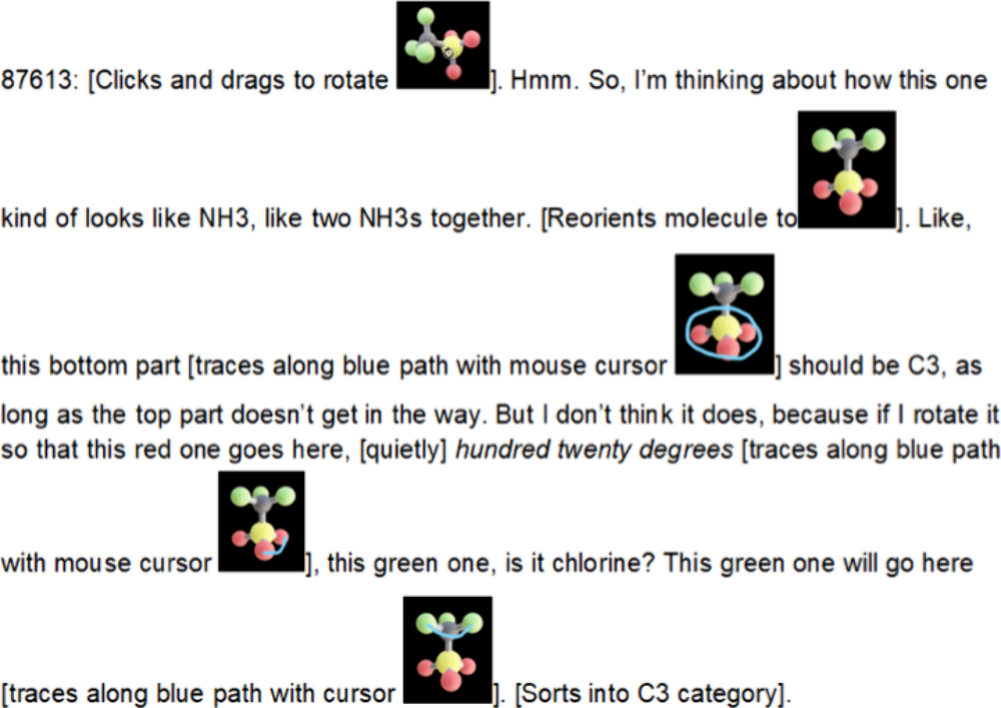
Example of a transcript excerpt from virtual interview. In this example, a student is engaging with the molecule benzotrifuroxan. The transcript is annotated with both verbal descriptions of their gestures (e.g., “traces along blue path with cursor”) as well as screen captures of molecular orientation and student inscriptions upon the molecule.

To enable meaningful contributions, the data can be analyzed from the perspective of both fine-grained knowledge elements and broader grouping strategies. For example, a researcher could use students’ verbal descriptions, cursor gestures, and inscriptions to infer specific elements of conceptual understanding related to molecular symmetry. In Figure 4, one might examine how the student coordinates verbal references to rotational angles (e.g., “approximately 120 degrees”), cursor movements that trace paths of atomic displacement, and on-screen annotations to assess their understanding of 3-fold rotational symmetry. A researcher might analyze whether the students’ manipulation aligns with formal symmetry operations (e.g., a *C*_3_ rotational axis), or whether their reasoning instead reflects an intuitive grasp of molecular symmetry. These multimodal signals (gesture, spatial reasoning, and verbal description) could be triangulated to provide insight into how students understand and apply symmetry operations. Such inferences would necessarily be contextual and theory-laden, but Figures 4 and 5 illustrate how the 3D card sorting environment affords opportunities for future work to make such knowledge visible and analyzable.

To support interpretive analysis of final groupings, researchers could explore how participants grouping rationales reflect broader epistemological orientations and conceptual resources. As shown in Figure 6, students organized molecular models using a range of strategies, including point group assignments, shape and complexity cues, or functional characteristics. A researcher might draw on framing constructs—e.g., Hammer et al.’s definition of framing as “a set of expectations an individual has about the situation···” (p. 102)^68^—to infer how students understood the purpose of the card sorting task and what types of knowledge they treated as relevant. For example, a participant who grouped molecules based on surface-level shape descriptors might been seen as framing the task as a perceptual characterization, whereas another who used point group labels might be invoking formalized disciplinary categories. These final groupings could be triangulated with process data (gestures, spatial manipulation, or verbal cues) to illuminate how students activated and weighted different ideas before deciding what to represent. As with the fine-grained examples above, these interpretations would necessarily depend on theoretical commitments, but the final sorts produced within this multimodal 3D environment offer a visible and analyzable trace of students’ reasoning priorities.

**Figure 6:**
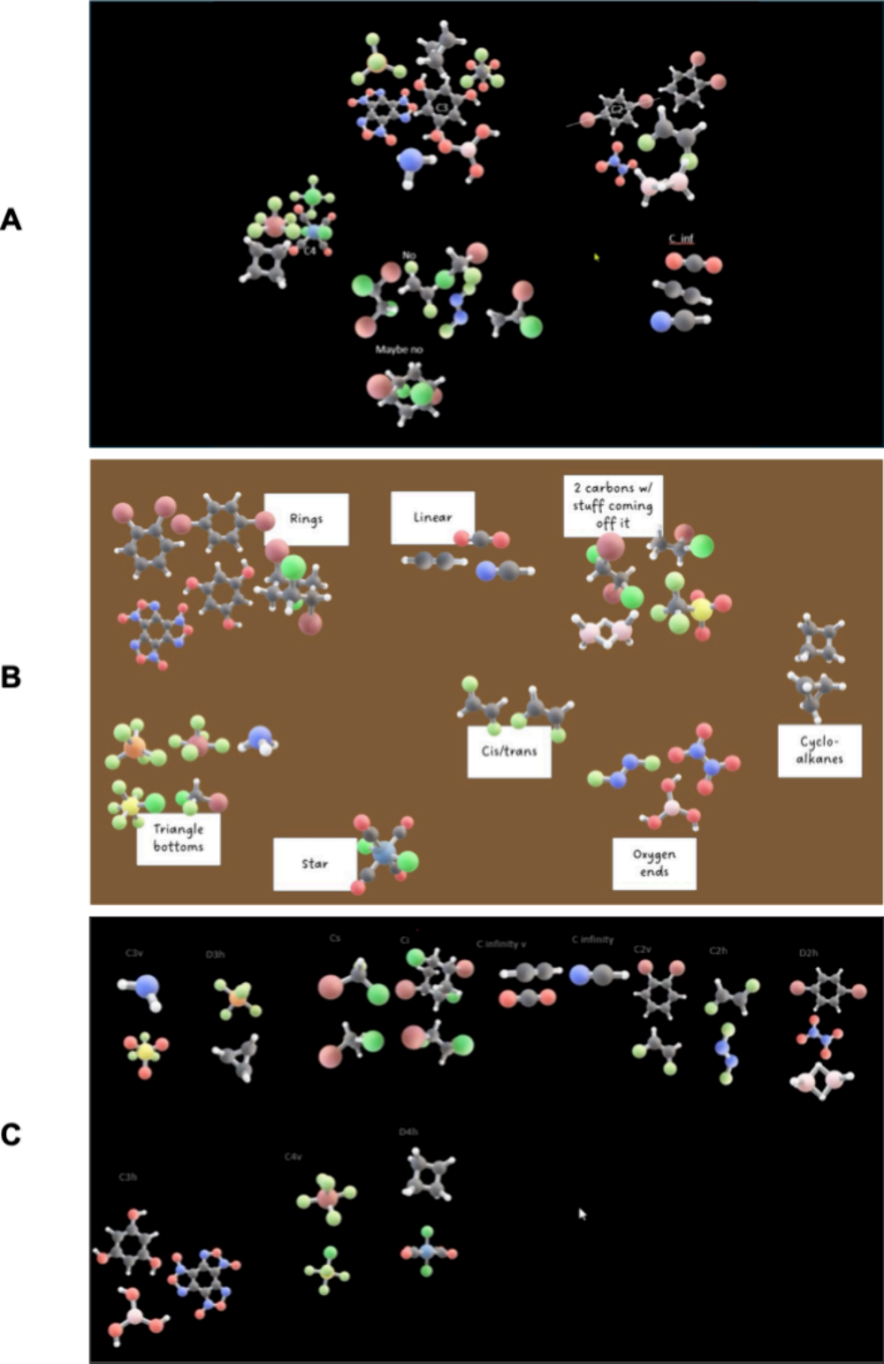
Examples of student-generated molecular symmetry categorizations from the card sorting task. (A) Undergraduate inorganic chemistry student organizing molecules based on the order of the principal axis of rotation; (B) general chemistry student grouping molecules by surface-level features (e.g., shape or perceived complexity); (C) undergraduate inorganic chemistry student categorizing molecules by point group. These final groupings illustrate variation not only in reasoning granularity and chemical knowledge, but also in how students framed the task, revealing distinct approaches to sorting molecules based on symmetry.

Finally, to address coherence among findings, we note that the variation in participants’ sorting strategies, gestures, and verbalizations is not treated as inconsistency or noise, but as a methodological affordance. The open-ended and multimodal design of the task surfaces diverse reasoning pathways, which enables interpretive synthesis of both fine-grained and course-grained insights. In this way, the design supports coherence not through uniformity of outcomes, but through a shared capacity to illuminate conceptual and epistemological dimensions of student thinking across cases.

## Limitations

While this design-focused contribution introduces 3D object-based card sorting as a novel method for eliciting spatial reasoning and symmetry-related ideas, we recognize important limitations that define the scope and transferability of our claims. Rather than reflecting flaws or gaps, our work’s limitations define the boundaries of interference.^69^ We chose to introduce and exemplify a method, not to generate empirical claims about student understanding. Readers should not interpret our findings as a definitive characterization of student learning or behavior. Future studies (including our own) can build on this work by applying the method in different instructional settings, disciplinary contexts, and with varied participant populations. We also invite other researchers to take up and adapt this approach to investigate reasoning about spatially complex or structurally nuanced chemical concepts. Such broader applications will be essential for evaluating the method’s generalizability, practical utility, and potential for instructional use.

Second, while we discuss cognitive and communicative affordances of 3D manipulable models, our study was not designed to systematically compare physical and virtual model formats. Participants interacted similarly with both types of models, and our examples highlight illustrative reasoning behaviors across both modalities. However, we cannot make claims about differential cognitive load or representational benefits associated with each format. These remain open empirical questions and opportunities for future comparative work.

Finally, like many qualitative, interview-based methodologies, our approach yields rich, multimodal data that can be time-intensive to collect, transcribe, and analyze. While this is a characteristic of in-depth qualitative work rather than a flaw, it may constrain the scalability of this method in large-sample contexts. Balancing the depth of insight with feasibility remains an important design consideration for future research using 3D object-based card sorting.

## Implications

### Research Implications

3D object-based card sorting is a promising methodology for studying spatially demanding concepts in chemistry. This approach can be adapted to investigate reasoning about molecular geometry, stereoelectronic effects, chirality, and stereochemistry. Its value lies not only in what it reveals about students’ spatial reasoning, but also in the rich, multimodal data it generates, enabling new research questions, analytic categories, and interpretations of conceptual activity.

Although gesture was not the primary focus of this study, the interview design created affordances for gestural reasoning to emerge. We encourage future work to explicitly examine gesture and to engage theoretical frameworks that support analysis of embodied cognition. Likewise, our use of both physical and virtual 3D models introduces opportunities to investigate how interaction modality shapes student engagement. Physical models afford spontaneous pointing and tactile manipulation; virtual models enable precision and layered digital annotations but may require additional interactional fluency. Future work should systematically explore these trade-offs, including effects on cognitive load, classification decisions, and reasoning strategies.

Taken together, these considerations point to the broader potential of 3D object-based card sorting as a flexible, extensible tool for CER. It offers opportunities to investigate how students reason across representations, enact ideas through physical interaction, and coordinate verbal, gestural, and manipulative modes of meaning-making. We invite researchers to adapt this approach in diverse contexts, further advancing its methodological contributions.

### Teaching Implications

Beyond its research applications, 3D object-based card sorting holds promise for classroom use. One long-term goal is to develop a formative assessment grounded in this method, one that enables instructors to elicit and interpret evidence of students’ prior knowledge and reasoning about molecular symmetry. Our approach aligns with the view that formative assessment is not defined by tools, but by processes that generate rich, interpretable data to inform instruction.^70^ By allowing students to demonstrate their understanding through gesture, manipulation, and categorization (not just verbal or written explanations) this methodology offers multiple, discipline-relevant modes of expression.

These features also resonate with principles of Universal Design for Learning,^71^ which call for multiple means of action and expression. When implemented in classrooms, the task can surface diverse forms of reasoning and promote multimodal dialogue among students. Whether used for formative assessment or collaborative learning, 3D object-based card sorting provides an inclusive and adaptable way to engage students with spatially complex chemical ideas in physical, digital, or hybrid environments.

## Conclusion

This work introduces 3D object-based card sorting as a novel, multimodal approach for investigating how students reason about molecular symmetry. By integrating both physical 3D-printed models and interactive virtual models, the approach elicits dimensions of student thinking that extend beyond verbal explanation, making reasoning visible across multiple modalities. A key insight from this work is the role of model interaction in supporting spatial reasoning. Behaviors such as exploratory rotation suggest that physical manipulation can reduce reliance on mental visualization, enabling students to externalize and refine their thinking. The opportunity to gesture, point, and annotate further supports the articulation and clarification of ideas, resulting in rich, multimodal data that capture reasoning in action. Although our focus was molecular symmetry, the method is adaptable to other chemistry topics involving spatial or representational complexity. It holds promise for developing inclusive formative assessments and for examining how students coordinate gesture, speech, and model-based reasoning in real time. By making students’ spatial reasoning strategies visible and analyzable, this work offers a generative methodological contribution to CER, one that opens new possibilities for investigating reasoning in spatially complex domains.

## Supplementary Material





## Notes

Virtual molecule sorting task (ZIP)
All .pdb files for the 25 molecules used in the sorting task (ZIP)
