# Supplementary material for: 3D Object-Based Card-Sorting: A Method for Eliciting Multimodal Reasoning in Chemistry
Source: J Chem Educ. 2025 Oct 3;102(10):4243–54. doi: 10.1021/acs.jchemed.5c00638 (PMC12529958; doi:10.1021/acs.jchemed.5c00638)
Supplement: Supplementary file 2 [file ed5c00638_si_002.zip › PDB Files for 3D Printing and Rendering/README_AuthorInfo.rtf]

3D Object-Based Card-Sorting: A Method for Eliciting Multimodal Reasoning in ChemistryRobin Morgenstern1, Samuel Pazicni1*, Sarah A. Swineheart1, and Maia Popova21Department of Chemistry, University of Wisconsin–Madison, Madison, Wisconsin 53706, United States2Department of Chemistry & Biochemistry, University of North Carolina at Greensboro, Greensboro, North Carolina 27412, United States Supporting Information
